# Deficiency of the exportomer components Pex1, Pex6, and Pex15 causes enhanced pexophagy in *Saccharomyces cerevisiae*

**DOI:** 10.4161/auto.28259

**Published:** 2014-03-18

**Authors:** James M Nuttall, Alison M Motley, Ewald H Hettema

**Affiliations:** Department of Molecular Biology and Biotechnology; University of Sheffield, Western Bank; Sheffield, UK

**Keywords:** exportomer, peroxin, peroxisome, pexophagy, Atg36, selective autophagy, Pex1, Pex6

## Abstract

Turnover of damaged, dysfunctional, or excess organelles is critical to cellular homeostasis. We screened mutants disturbed in peroxisomal protein import, and found that a deficiency in the exportomer subunits Pex1, Pex6, and Pex15 results in enhanced turnover of peroxisomal membrane structures compared with other mutants. Strikingly, almost all peroxisomal membranes were associated with phagophore assembly sites in *pex1*Δ* atg1*Δ cells. Degradation depended on Atg11 and the pexophagy receptor Atg36, which mediates degradation of superfluous peroxisomes. Mutants of *PEX1*, *PEX6,* and *PEX15* accumulate ubiquitinated receptors at the peroxisomal membrane. This accumulation has been suggested to trigger pexophagy in mammalian cells. We show by genetic analysis that preventing this accumulation does not abolish pexophagy in *Saccharomyces cerevisiae*. We find Atg36 is modified in *pex1*Δ cells even when Atg11 binding is prevented, suggesting Atg36 modification is an early event in the degradation of dysfunctional peroxisomal structures in *pex1*Δ cells via pexophagy.

## Introduction

Maintenance of cellular homeostasis requires that the quality of organelles is checked and controlled. Damaged or superfluous organelles are specifically marked for degradation by selective autophagy.^1^ Peroxisomes are ubiquitous organelles and are required for a variety of metabolic processes including fatty acid β-oxidation. In *S. cerevisiae*, peroxisomes are degraded when their metabolism becomes superfluous. This degradation is particularly evident under conditions of nitrogen starvation, when macroautophagy is induced.^2^ In the methylotrophic yeast *Hansenula polymorpha*, protein aggregates in peroxisomes of a mutant catalase variant are cleared by asymmetric peroxisome fission to separate the aggregate from the mother organelle, and the aggregate-containing organelles are subsequently degraded by autophagy.^3^ Likewise, selective removal of Pex3 from peroxisomes or exposure to excessive ROS induces pexophagy in this yeast.^4^ However, the machinery required for the recognition of damaged peroxisomes remains unknown.

Pexophagy is a selective form of autophagy. In *S. cerevisiae* it relies on the receptor Atg36 that is bound to peroxisomes via the intergral peroxisomal membrane protein Pex3.^5^ Atg36 also physically interacts with the scaffold protein Atg11 that is commonly used in selective forms of autophagy to link cargo receptors with the core autophagy machinery.^6^^,^^7^ Atg36 also interacts with Atg8.^5^^,^^8^

Mutations that affect Atg36 interaction with Atg11 and Atg8 have been described.^8^ Whereas the former blocks nitrogen starvation-induced pexophagy, the latter only delays it.^8^ Atg36 is modified differentially during nitrogen starvation compared with normal growth conditions. In this respect, Atg36 resembles other cargo receptors for selective autophagy in yeasts, in that they all interact with Atg11 and Atg8. Studies on the mitophagy receptor Atg32, and the pexophagy receptors of *Pichia pastoris* (Atg30) and *S. cerevisiae* (Atg36) suggest that these interactions are regulated by posttranslational modifications (phosphorylation) and this is thought to modulate autophagy flux.^8^^-^^12^

Peroxisomal matrix proteins are posttranslationally imported from the cytosol. Most proteins contain a conserved C-terminal tripeptide, the peroxisomal targeting signal type 1 (PTS1) that is recognized by the cytosolic receptor Pex5. The receptor cycle involves cargo recognition in the cytosol, membrane docking of cargo-receptor complex, cargo release, and recycling of receptor (for a review, see ref. 13). In vitro and in vivo data implicate Pex1 and Pex6 in Pex5 recycling.^14^^-^^16^ Pex1 and Pex6 are required only for the ATP-dependent step of the receptor cycle, release of Pex5 back to the cytosol.^16^^,^^17^ Pex1, Pex6, and their peroxisomal membrane anchor, Pex15, form a subcomplex that is part of the export machinery that collectively has been termed ‘exportomer.’^13^

The Pex5 receptor cycle is regulated by Pex5 ubiquitination and deubiquitination.^18^^-^^22^ Cells deficient in any of the exportomer subunits—Pex1, Pex6, or Pex15—accumulate ubiquitinated Pex5 at the peroxisomal membrane.^16^^,^^17^^,^^20^ The PTS2 receptor and its co-receptors have been proposed to undergo a similar recycling route.^23^ In human cells, ubiquitinated Pex5 accumulation at the peroxisomal membrane has been suggested to be a signal for pexophagy via binding by the ubiquitin-binding protein NBR1,^24^ and expression of a PEX3-ubiquitin fusion induces pexophagy in mammalian cells.^25^ However, ubiquitinated Pex5 is degraded by the proteasome in wild-type (WT) yeast and not in the vacuole.^19^ Furthermore, whereas cargo recognition via ubiquitin has been widely documented in higher eukaryotes,^26^^,^^27^ there is no evidence for such a role for ubiquitin in *S. cerevisiae*.

Various peroxins have been implicated in pexophagy.^4^^,^^5^^,^^9^^,^^28^^-^^30^ Since peroxisome formation and turnover are intimately linked processes, we tested whether a deficiency in peroxisomal protein import results in altered pexophagy. We found that deletion of *PEX1*, *PEX6,* or *PEX15* leads to increased pexophagy under all conditions compared with peroxisome turnover in other import mutants or WT cells. This turnover was dependent on Atg11 and Atg36 and was specific to peroxisomal structures, as mitophagy and nonselective macroautophagy were not induced. Increased pexophagy is not due to accumulation of PTS receptors at the peroxisomal membrane in *pex1∆* cells. Interestingly, Atg36 is differentially modified in *pex1Δ* cells compared with WT cells. Inducible removal of Pex1 by degron tagging suggests that Pex1 is not regulating pexophagy directly but rather that its long-term absence results in dysfunctional peroxisomal structures that are marked for degradation.

## Results

### Increased turnover of peroxisomal structures in *pex1∆*, *pex6∆,* and *pex15∆* mutants

Most peroxisome assembly mutants import membrane proteins but fail to import lumenal proteins into peroxisomal membrane structures. The number of peroxisomal remnants varies between mutants. We expressed the membrane protein Pex11-GFP under various conditions and found that *pex1∆*, *pex6∆,* and *pex15∆* mutants had very few membrane structures compared with other import mutants. This difference was especially obvious in glucose cultures grown past logarithmic phase and also under nitrogen-starvation conditions, when most *pex1∆*, *pex6∆,* and *pex15∆* cells lacked any detectable puncta of Pex11-GFP (Fig. 1C; Fig. S1A). A faint vacuolar labeling was often visible in these cells, and this is reminiscent of Pex11 breakdown via pexophagy.^5^ Peroxisomal membranes were present in most *pex1∆*, *pex6∆,* and *pex15∆* cells grown on oleate medium (Fig. S1A), a condition that induces proliferation of peroxisomes.

**Figure:**
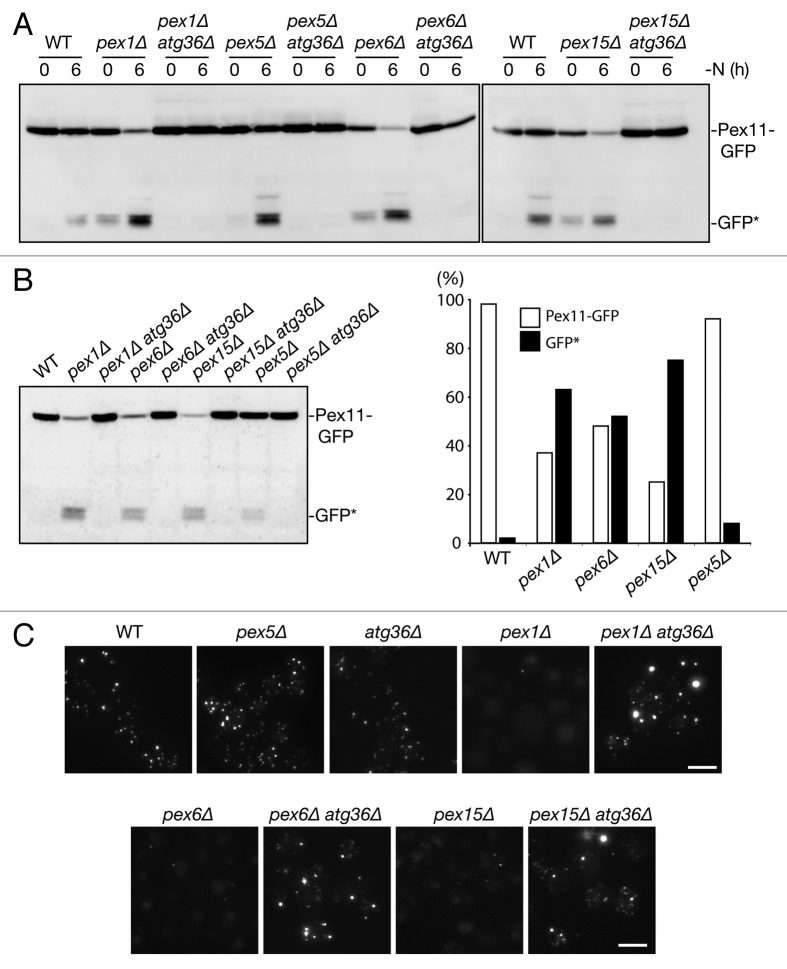
**Figure 1.***pex1∆*, *pex6∆,* and *pex15∆* cells show increased pexophagy that is dependent on the pexophagy receptor Atg36. (**A**) Oleate grown cells (0) were shifted to nitrogen starvation conditions for 6 h (6) and pexophagy was monitored by Pex11-GFP breakdown. GFP* indicates the relatively protease-resistant degradation product and reflects vacuolar breakdown. Increased pexophagy is blocked in double mutants with *ATG36*. Pexophagy was monitored in post-logarithmic cultures and analyzed with Pex11-GFP immunoblotting using monoclonal anti-GFP antibody (**B**) or fluorescence microscopy (**C**). The full-length and breakdown product signals in post-logarithmic cultures (**B**) were quantified in ImageJ and expressed as percentage of total signal. Scale bar: 5 µm.

To monitor pexophagy, we followed Pex11-GFP breakdown after shifting from oleate to glucose medium lacking a nitrogen source. Accumulation of the relatively protease-resistant GFP is indicative of vacuolar breakdown of Pex11-GFP.^5^ Under these pexophagy-inducing conditions, *pex1∆*, *pex6∆,* and *pex15∆* cells have increased breakdown of Pex11-GFP compared with other mutants defective in import of peroxisomal lumenal proteins (Fig. 1A; Fig. S1B). Even under peroxisome proliferation conditions (Fig. 1A, t = 0) we observed a low level of Pex11-GFP breakdown in *pex1∆*, *pex6∆,* and *pex15∆* cells, whereas this does not occur in WT cells. *pex5∆* cells show more degradation than WT cells after 6 h starvation (Fig. 1A), but comparison of the amount of full-length Pex11-GFP shows the degradation is relatively little compared with that in *pex1∆*, *pex6∆,* and *pex15∆* cells. Enhanced degradation of Pex11-GFP was also observed in post-logarithmic *pex1∆*, *pex6∆,* and *pex15∆* cultures (Fig. 1B; Fig. S1C). Quantification of full-length and breakdown products of Pex11-GFP under this condition confirmed that pexophagy was enhanced in these mutants compared with WT and *pex5∆* cells (Fig. 1B; Fig. S1C). Deletion of *ATG36*, the gene encoding the pexophagy receptor, blocked breakdown of Pex11-GFP in all strains, under both conditions (Fig. 1A and B), and increased the number of Pex11-GFP labeled structures (Fig. 1C). We conclude that in *pex1∆*, *pex6∆,* and *pex15∆* cells the low number of peroxisomal membrane structures is a consequence of increased pexophagy.

### Pexophagy is selectively induced in *pex1∆* cells

We tested the effect of *PEX1* deletion on other forms of autophagy. WT and *pex1∆* cells expressing the mitochondrial outer membrane protein Om45-GFP were grown on glycerol medium for up to 55 h.^31^^,^^32^ Mitophagy was measured in post-logarithmic cells by degradation of Om45 and the appearance of free GFP indicating vacuolar degradation. The typical GFP breakdown product appeared with similar kinetics in *pex1∆* compared with WT cells (Fig. 2B). Although *pex1∆* cells did not induce mitophagy until 24 h after switching to glycerol medium, pexophagy occurred in *pex1∆* cells growing logarithmically on glycerol medium (Fig. 2A). Nonselective autophagy was tested using the Pho8∆60 vacuolar activation assay.^33^ We found the level of nonselective autophagy in post-logarithmic glucose-grown *pex1∆* cultures was low, similar to that in WT cells, and was induced upon nitrogen starvation (Fig. 2C). Since mitophagy and nonselective autophagy are not increased in *pex1∆* cells, we conclude that a defect in Pex1 results in the selective autophagy of peroxisomal membranes.

**Figure:**
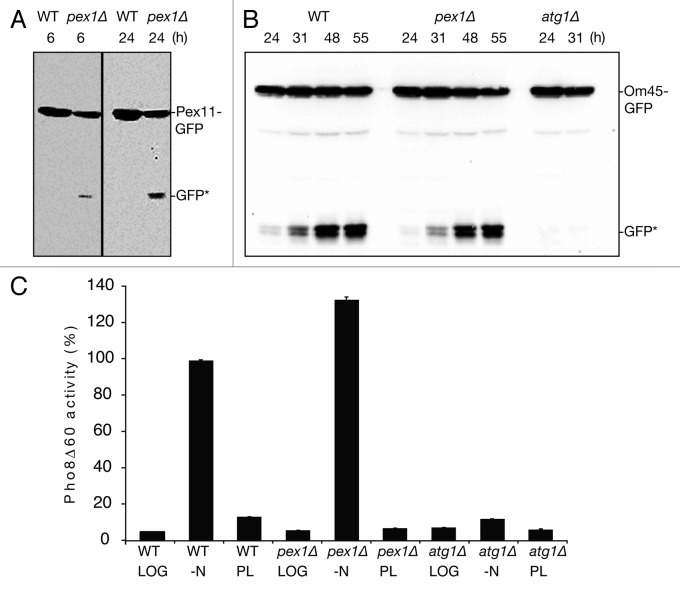
**Figure 2.** Nonselective macroautophagy and mitophagy are not affected in *pex1∆* mutants. (**A**) Pexophagy as assayed by Pex11-GFP breakdown in WT and *pex1∆* cells grown for 6 or 24 h on glycerol medium. Immunoblotting was done with monoclonal anti-GFP antibody. (**B**) Mitophagy was assayed by Om45-GFP breakdown after growing WT, *pex1∆,* and *atg1∆* cells for the times indicated on glycerol medium. Immunoblotting was done with monoclonal anti-GFP antibody. (**C**) WT, *pex1∆,* and *atg1∆* cells were assayed for nonselective macroautophagy by the alkaline phosphatase assay. Logarithmically growing (LOG), nitrogen starved (-N) and post-logarithmic growing cultures (PL) were collected and processed for Pho8∆60 activity. The results represent the mean and standard deviation (SD) of an experiment done in triplicate. WT 4 h starvation is set at 100%.

### Strongly enhanced association of peroxisomal membranes with the PAS in *pex1∆ atg1∆* cells requires Atg36

In *atg1∆* cells, phagophores form but further maturation into autophagosomes is blocked.^34^ Consequently, many components of the autophagic machinery and cargoes localize to the phagophore assembly site (PAS) in *atg1∆* cells under autophagy-inducing conditions. Peroxisomes cluster to the PAS (labeled by GFP-Atg11) in 65% of *atg1∆* cells grown under nitrogen-starvation conditions.^5^ When examining the proximity of Atg11 with peroxisomal membranes in *atg1∆* cells under vegetative growth conditions (Fig. 3A), many cells contain a peroxisome adjacent to the Atg11-labeled PAS. However, most *atg1∆* cells under this condition contain at least 10 peroxisomes per cell, most of which are not associated with the PAS. In *pex1∆ atg1∆* cells, Pex11-mRFP was present in a reduced number of brightly fluorescent structures, and here we found the frequency of colocalization was strikingly increased compared with *atg1∆* cells: almost all (> 95%) Pex11-mRFP-labeled structures were associated with or in close proximity to GFP-Atg11. The association between Pex11 and Atg11 was increased in *pex1∆ atg1∆* cells compared with a control mutant, *pex4∆ atg1∆*, and was dependent on Atg36 (Fig. 3A), suggesting that peroxisomes in *pex1∆ atg1∆* cells were 'primed' for degradation. The observation that peroxisomes are dispersed in *pex1∆ atg36∆* cells suggests the clustering also requires the presence of Atg36.

**Figure:**
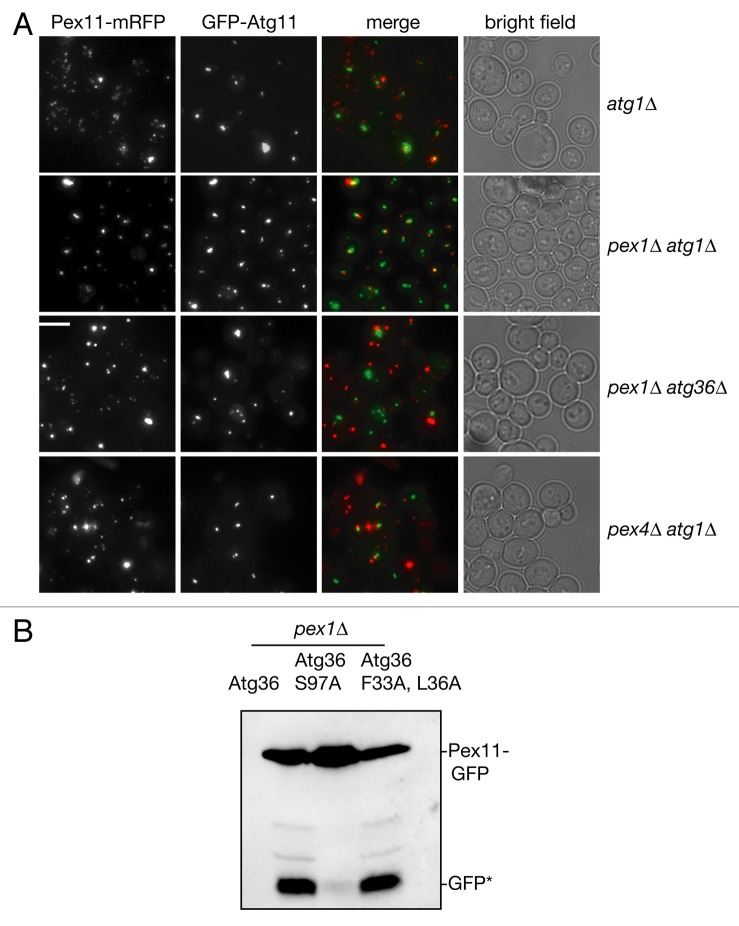
**Figure 3.** Pexophagy in post-logarithmic *pex1∆* cultures requires interaction of Atg36 with Atg11. (**A**) Fluorescence microscopy of strains expressing Pex11-mRFP and GFP-Atg11 as indicated were grown to post-logarithmic phase in glucose medium. Scale bar: 5 µm. (**B**) Pex11-GFP pexophagy analysis of *pex1∆* cells expressing different alleles of *ATG36:* WT *ATG36*, *ATG36*-*S97A* or *ATG36*-*F33A*, *L36A*. Cells were grown for 24 h on glucose medium and analyzed by immunoblotting using monoclonal anti-GFP antibody.

Nitrogen starvation-induced pexophagy depends on the adaptor protein Atg11 that links the pexophagy receptor Atg36 to the core autophagy machinery.^5^ The breakdown of Pex11-GFP in *pex1∆* cells was found to be dependent upon Atg11 under all growth conditions tested (Fig. S2). A mutation in Atg36 that blocks the interaction with Atg11 also blocked pexophagy in *pex1∆* cells (Fig. 3B).^8^ Conversely, a mutation reported to block interaction of Atg36 with Atg8 did not block pexophagy (Fig. 3B).^8^ We conclude that association of peroxisomal structures with the PAS in *pex1∆* cells is dependent on Atg36, and that their breakdown requires Atg36 interaction with Atg11.

### Depletion of Pex1 triggers pexophagy

The enhanced pexophagy in *pex1∆* cells may reflect either a direct role of Pex1 in regulating pexophagy, or an indirect role, with the Pex1 deficiency resulting in aberrant peroxisomes that are subsequently degraded. To distinguish between these possibilities, we created a conditional allele of Pex1 by tagging it with the auxin-inducible HA-degron (aid) tag.^35^ Within an hour of auxin addition, Pex1-HA-aid was degraded to undetectable levels (Fig. 4A). Depletion of Pex1 should rapidly block peroxisomal matrix protein import, and this was indeed the case, with import being blocked when cells were examined 90 min after auxin addition (Fig. 4B).

**Figure:**
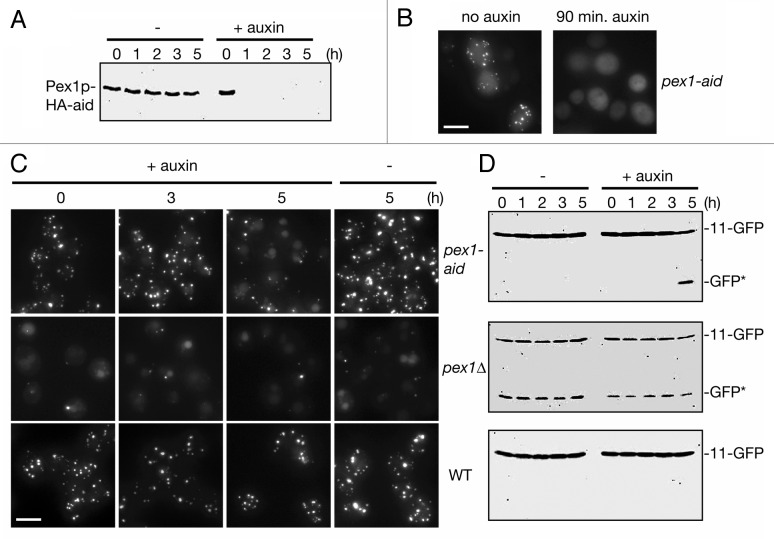
**Figure 4.** Degradation of Pex1 causes an import defect before it causes pexophagy. (**A**) Cells containing Pex1 tagged at the C terminus with an auxin-inducible degron-HA tag were grown overnight on glucose medium and treated with (+) or without (−) auxin (500 μM). Samples were collected at the times indicated and analyzed by immunoblotting using monoclonal anti-HA antibody. (**B**) Peroxisomal import of matrix protein marker GFP-PTS1 was assessed after 90 min in the presence (right hand panel) or absence (left hand panel) of 500 μM auxin. Cells were grown to logarithmic phase on raffinose, and GFP-PTS1 was induced by switching cells for 30 min to galactose medium followed by a 30 min chase in glucose. Scale bar: 5 µm. (**C**) Pexophagy as assessed by disappearance of Pex11-GFP puncta occurs in Pex1-HA-aid cells 3–5 h after auxin addition. Scale bar: 5 µm. (**D**) Cells in (**C**) were analyzed by immunoblotting using monoclonal anti-GFP antibody.

We followed pexophagy in Pex1-HA-aid depleted cells by fluorescence microscopy and western blotting of Pex11-GFP (Fig. 4C and D). Whereas import was already blocked within 90 min of auxin addition, pexophagy became apparent 3–5 h after auxin addition, with peroxisome number decreasing and vacuolar fluorescence appearing (Fig. 4C). Peroxisomal membrane structures were completely absent in approximately 5% of the cells 5 h after auxin treatment. The drastic reduction of peroxisomal membrane structures after auxin addition indicates existing peroxisomal structures are rapidly broken down. These findings were confirmed by immunoblotting, with Pex11-GFP breakdown being evident 5 h after auxin addition (Fig. 4D). No pexophagy was evident in control cells grown under the same conditions, whereas *pex1∆* cells displayed Pex11-GFP breakdown throughout the time course of the experiment. We conclude that peroxisomes are degraded only after prolonged depletion of Pex1.

### Accumulation of PTS receptors on peroxisomes is not required for enhanced pexophagy

Pex1, Pex6, and Pex15 are required for the extraction and export of the PTS1 receptor Pex5 from peroxisomes. A defect in Pex5 extraction results in its accumulation at the peroxisomal membrane in a polyubiquitinated form. It has been suggested that accumulation of polyubiquitinated receptors may be a signal for pexophagy in mammalian cells.^25^ *pex1∆* mutants deficient in all peroxisomal import receptors (PTS1 receptor, Pex5, and PTS2 coreceptors, Pex18 and Pex21), still showed enhanced pexophagy in post-logarithmic cultures (Fig. 5A and B) and during nitrogen starvation (Fig. 5C). We conclude that accumulation of import receptors at the peroxisomal membrane is not the trigger for pexophagy in *S. cerevisiae*.

**Figure:**
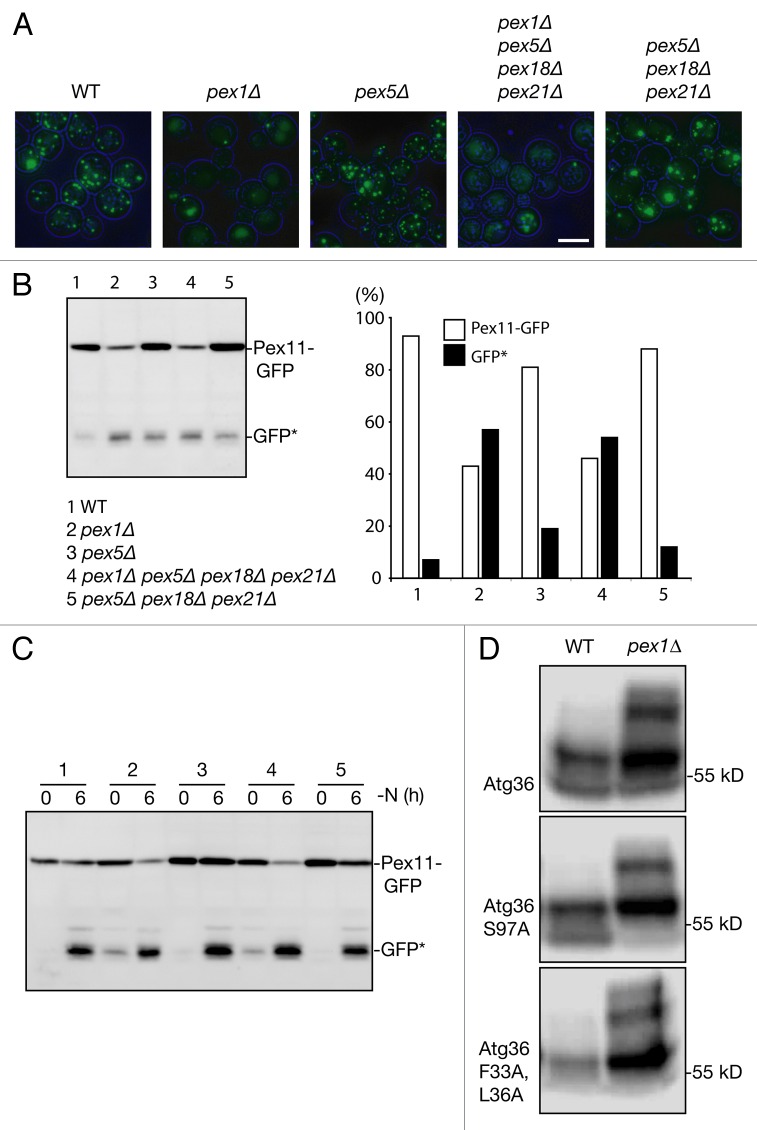
**Figure 5.** Pexophagy is not triggered by accumulation of import receptors at the peroxisomal membrane. Pex11-GFP was expressed in strains as indicated. Cells were grown to post-logarithmic phase and examined for Pex11-GFP fluorescence (**A**) or immunoblotting using monoclonal anti-GFP antibody (**B**). Scale bar: 5 µm. The full-length and breakdown product signals in post-logarithmic cultures (B) were quantified in ImageJ and expressed as percentage of total signal. Oleate-grown cells (0) were shifted to nitrogen-starvation conditions for 6 h (6) and pexophagy was assessed by Pex11-GFP breakdown using monoclonal anti-GFP antibody (**C**). (**D**) The indicated *ATG36-protein A* alleles in WT and *pex1∆* cells were grown overnight on glucose and analyzed by immunoblotting using PAP antibody.

### Atg36 is differentially modified in *pex1∆* cells compared with WT cells

Mechanisms regulating selective autophagy are poorly characterized. We observed Atg36 migrating differently on SDS-PAGE in *pex1∆* cells under nonstarvation conditions compared with WT cells. The pattern of migration of Atg36 in *pex1∆* cells was not affected in Atg36 S97A or Atg36 F33A, L36A mutants,^8^ indicating modification of Atg36 is independent of its interaction with Atg11 and Atg8, respectively (Fig. 5D).

## Discussion

On examining a group of 12 peroxisomal import mutants, we noticed a striking reduction in numbers of peroxisomal structures in the AAA ATPase mutants, *pex1∆*, *pex6∆,* and *pex15∆*, and found this to be a consequence of pexophagy. Peroxisomal structures in these mutants were broken down at low levels under conditions where there was no pexophagy in WT cells. Breakdown was further increased under conditions that stimulate pexophagy, resulting in an almost complete absence of peroxisomal structures during nitrogen starvation. The strikingly increased proximity of peroxisomal structures with the PAS in *pex1∆ atg1∆* cells suggests that all peroxisomal membrane structures in these cells are primed for degradation.

What could be the role of Pex1, Pex6, and Pex15 in protecting peroxisomes from turnover? These peroxins have a well-established role in peroxisomal matrix protein import. Turnover of peroxisomal membrane structures is increased in these mutants compared with other mutants where import is blocked, indicating that an import defect per se is not the trigger for peroxisome degradation. It is important to understand what sets these mutants apart from other import mutants particularly because most of the patients suffering from peroxisome assembly disorders have mutations in Pex1, Pex6, or the Pex15 ortholog Pex26.^36^ We examined the timing of pexophagy after induced Pex1 degradation. Pexophagy does not occur until several hours after a block in import is evident. This delay suggests accumulation or depletion of a regulator before pexophagy is triggered.

Ubiquitination is important in selective autophagy in mammalian cells.^26^^,^^27^^,^^37^ In *pex1∆*, *pex6∆,* and *pex15∆* mutants, the PTS receptor recycling pathway is blocked and Pex5 accumulates in ubiquitinated forms at the peroxisomal membrane.^16^^,^^17^ The ubiquitin-binding autophagy receptor NBR1 contributes to pexophagy in mammalian cells and it has been suggested that ubiquitination of Pex5 is a trigger for pexophagy.^24^ However, cargo ubiquitination appears not to play a role in selective autophagy in yeast.^26^ In support of this, when we prevent accumulation of ubiquitinated PTS receptors by constructing strain *pex1∆ pex5∆ pex18∆ pex21∆*, pexophagy continues unaffected. Therefore, accumulation of ubiquitinated PTS receptors is not the signal for pexophagy in *S. cerevisiae pex1∆* cells.

Another role that has been proposed for *S. cerevisiae* Pex1 and Pex6 is in maturation of newly forming peroxisomes. Our depletion studies indicate that pre-existing peroxisomes are degraded upon depletion of Pex1. It is difficult to envisage how a role of Pex1 and Pex6 in de novo peroxisome formation would affect existing peroxisomes, and we consider it unlikely that this proposed function of Pex1 and Pex6 explains the increased turnover of peroxisomes in *pex1∆* and *pex6∆* cells. The low number of peroxisomal structures in *pex1∆* and *pex6∆* cells was proposed to be due to an early block in peroxisome formation before structures multiply by fission.^38^ The conclusions that can be drawn from these experiments should be reevaluated in light of our finding of increased turnover of peroxisomal membrane structures in these mutants. For instance, we show *pex1∆* and *pex6∆* cells have multiple peroxisomal structures when pexophagy is blocked (in the absence of Atg36). This indicates that the low number of peroxisomal membranes results from decreased stability and not a defect in peroxisomal membrane formation.

It is possible that the ability to protect peroxisomes from degradation reflects a function of these exportomer subunits independent from their role in the receptor cycle. Pex15 recruits Pex1 and Pex6, which are both AAA-ATPases that act to release Pex5 from the peroxisomal membrane.^16^ One possibility is that they have substrates in addition to the PTS receptors. Depletion of Pex1 may result in a gradual accumulation of these substrates, which triggers pexophagy by stimulating Atg36 modification. Unfortunately, technical difficulties have thus far prevented us from determining the exact nature of the modification of Atg36.

The machinery required for pexophagy in *pex1∆*, *pex6∆,* and *pex15∆* cells shares factors with that required during nitrogen-starvation, with Atg11 linking peroxisomal membranes to the core autophagy machinery. Pexophagy in *pex1∆* cells is blocked when the Atg11-binding motif of Atg36 is mutated, underlining the importance of this interaction. The finding that Atg36 is differently modified in *pex1∆* cells even when its Atg11-binding motif is mutated suggests Atg36 modification precedes Atg11 binding and supports our hypothesis that this is the trigger for pexophagy. In contrast, pexophagy in *pex1∆* cells is unaffected when the Atg8-binding motif of Atg36^8^ is mutated, providing support to the finding that Atg8 binding is not a major contributor to receptor-mediated delivery of peroxisomes^8^^,^^39^ or mitochondria^11^ to the PAS in *S. cerevisiae*.

## Materials and Methods

### Yeast strains, media, and growth conditions

The yeast strains used in this study are listed in Table 1. Gene tagging and disruptions were performed by homologous recombination and strains were checked by PCR. For all experiments, cells were grown overnight in selective medium containing glucose. For analysis of phenotypes by microscopy, cells were subsequently diluted to 0.1 OD_600_ in fresh selective glucose medium and grown for 2 to 3 cell divisions (4–6 h), prior to imaging. Where the induction of a reporter protein was required, cells were transferred to selective medium containing galactose (Sigma-Aldrich, G0625) at 0.1 OD_600_ and grown for the time indicated in the figures and text. Yeast cells were grown at 30 °C in either of the following media: rich YPD medium (1% yeast extract, 2% peptone, 2% glucose), selective medium (YM2) for the selection of the uracil prototrophic marker (carbon source, 0.17% yeast nitrogen base without amino acids and ammonium sulfate, 0.5% ammonium sulfate, 1% casamino acids) or selective medium (YM1) for the selection of all prototrophic markers (carbon source, 0.17% yeast nitrogen base without amino acids and ammonium sulfate, 0.5% ammonium sulfate). Regarding the carbon sources, glucose and galactose were added to 2% (w/v) and glycerol to 3% (v/v). In all, 5–10 OD_600_ units were collected at selected time points as indicated in the figures and text. The vacuolar membrane was stained with FM 4-64 (Molecular Probes, T-13320) as previously described.^42^ Auxin yeast strains were based on previously published strains^35^ and obtained from the Yeast Genetic Resource Centre, Osaka University (http://yeast.lab.nig.ac.jp/nig/index_en.html; BY25594).

**Table:** **Table 1.** Yeast strains used in this study

Strain and genotype	Reference
BY4741 MATa *his3-1 leu2-0 met15-0 ura3-0*	Euroscarf
BY4742 MATα *his3-1 leu2-0 lys2-0 ura3-0*	Euroscarf
C13 abys 86 MATα *ura3∆5 leu2-3 112 his3 pra1-1 prb1-1 prc1-1 cps1-3*	40
BY4741 *pex1∆::kanMX4*	Euroscarf
BY4741 *pex2∆::kanMX4*	Euroscarf
BY4741 *pex4∆::kanMX4*	Euroscarf
BY4741 *pex5∆::kanMX4*	Euroscarf
BY4741 *pex6∆::kanMX4*	Euroscarf
BY4741 *pex7∆::kanMX4*	Euroscarf
BY4741 *pex8∆::kanMX4*	Euroscarf
BY4741 *pex10∆::kanMX4*	Euroscarf
BY4741 *pex12∆::kanMX4*	Euroscarf
BY4741 *pex13∆::kanMX4*	Euroscarf
BY4741 *pex14∆::kanMX4*	Euroscarf
BY4741 *pex15∆::kanMX4*	Euroscarf
BY4741 *pex17∆::kanMX4*	Euroscarf
BY4741 *pex19∆::kanMX4*	Euroscarf
BY4741 *pex22∆::kanMX4*	Euroscarf
BY4741 *atg36∆::kanMX4*	Euroscarf
BY4741 *atg1∆::kanMX4*	Euroscarf
BY4741 *atg8∆::kanMX4*	Euroscarf
BY4741 *atg11∆::kanMX4*	Euroscarf
BY4741 *atg1∆::kanMX4 pex1∆::HIS5*	This study
BY4741 *atg8∆::kanMX4 pex1∆::HIS5*	This study
BY4741 *atg11∆::kanMX4 pex1∆::HIS5*	This study
BY4741 *pex1∆::kanMX4 atg36∆::HIS5*	This study
BY4741 *pex5∆::kanMX4 atg36∆::HIS5*	This study
BY4741 *pex6∆::kanMX4 atg36∆::HIS5*	This study
BY4741 *pex15∆::kanMX4 atg36∆::HIS5*	This study
BY4741 *pex4∆::kanMX4 atg1∆::HIS5*	This study
BY4741 *pex5∆::kanMX4 pex18∆::LEU2 pex21∆::hphMX4*	This study
BY4741 *pex5∆::kanMX4 pex1∆::HIS5 pex18∆::LEU2 pex21∆::hphMX4*	This study
BY4741 *OM45-GFP::hphMX4*	5
BY4741 *atg1∆::kanMX4 OM45-GFP::hphMX4*	5
BY4741 *pex1∆::HIS5 OM45-GFP::hphMX4*	This study
BY4741 *pho13∆::MET15 ∆pho8::pho8 60-LEU2*	41
BY4741 *atg1∆::kanMX4 pho13∆::MET15 pho8∆::pho8 60-LEU2*	41
BY4741 *pex1∆::URA3 pho13∆::MET15 pho8∆::pho8 60-LEU2*	This study
C13 abys 86 *ATG36-PtA::HIS5*	This study
C13 abys 86 *atg36∆::URA3-PtA::HIS5*	This study
C13 abys 86 *atg36∆::URA3-PtA::HIS5 pex1∆::LEU2*	This study
*pex1∆::kanMX4 pex1∆::LEU2 atg36∆::HIS5 ATG36-PtA::HIS5*	This study
*pex1∆::kanMX4 pex1∆::LEU2 atg36∆::HIS5 ATG36 S97A-PtA::HIS5*	This study
*pex1∆::kanMX4 pex1∆::LEU2 atg36∆::HIS5 ATG36 F33A L36A-PtA::HIS5*	This study
BY4742 *pex1∆::kanMX4*	Euroscarf
BY4742 *pex1∆::kanMX4 PEX11-GFP::HIS5*	This study
W303-1A MATa *ura3-1::ADH1-OsTIR1-9Myc(URA3) ade2-1 his3-11 15 leu 2-3 112 trp1–1 can1–100*	35
W303-1A MATa *ura3-1::ADH1-OsTIR1-9Myc(URA3) ade2-1 pex1∆::HIS5 15 leu 2-3 112 trp1-1 can1-100*	This study
W303-1A MATa *ura3-1::ADH1-OsTIR1-9Myc(URA3) ade2-1 his3-11 15 leu 2-3 112 trp1-1 can1-100 PEX1-3HA-IAA17::hphMX4*	This study

### Construction of strains and plasmids

Yeast expression plasmids were based on the parental plasmids ycplac33 and ycplac111.^43^ The majority of constructs used in this study were generated by homologous recombination in yeast.^44^ The ORF of interest was amplified by PCR. The 5′ ends of the primers included 18 nucleotide extensions homologous to plasmid sequences flanking the intended insertion site, to enable repair of gapped plasmids by homologous recombination. For expression of genes under control of their endogenous promoter, 500 nucleotides upstream from the ORF were included. Galactose-inducible constructs contained the *GAL1* and *GAL10* intragenic region. All yeast constructs contain the *PGK1* terminator. Oleate-inducible GFP-PTS1 is controlled by the peroxisomal catalase (*CTA1*) promoter.

To tag Pex1-HA-aid in the genome for auxin experiments, a modified version of pSM409^35^ was created where a 3xHA tag was recombined at the N terminus of IAA17 using SalI and KpnI. Genomic tagging of Pex11-GFP was performed using recombination based on pFA6a-GFP(S65T)-HIS3MX6.^45^

Genomic point mutants were engineered into a vector containing Atg36 under the control of its own promoter using site-directed mutagenesis. These were integrated by homologous recombination into a C13 abys 86 yeast strain^40^ where Atg36 was replaced with the *URA3* cassette and tagged with protein A. Positive colonies were screened for by selection on 5-FOA (Melford, F5001).^46^ Resulting strains were mated with BY4741 or BY4742 derivatives and diploids selected for to account for the vacuolar deficiency of the C13 abys 86 strain.

### Assays for pexophagy, mitophagy, and macroautophagy

For peroxisome induction, cells were transferred to oleate medium (YM2 oleate: YM2 plus 0.12% oleate [v/v], 0.2% Tween-40^®^ [v/v; Sigma-Aldrich, P1504], 0.1% yeast extract) at a 1/10 overnight dilution. Pexophagy was induced by transferring cells to starvation medium lacking a nitrogen source (SD-N; 0.17% yeast nitrogen base without amino acids and ammonium sulfate, 2% glucose). Mitophagy was followed using the Om45-GFP processing assay as described previously.^47^ For monitoring macroautophagy, the alkaline phosphatase activity of Pho8∆60 was measured as described previously and performed in triplicate.^33^

### Immunoblotting

Immunoblotting was performed as described previously.^5^ Tagged proteins were detected using either monoclonal anti-HA (Sigma-Aldrich, H9658), monoclonal anti-GFP (Roche, 11814460001) or peroxidase-anti-peroxidase (PAP) (Sigma-Aldrich, P1291). Secondary antibody was HRP-linked anti-mouse polyclonal (Bio-Rad, 1706516). Detection achieved using enhanced chemiluminescence (Biological Industries, 20-500) and chemiluminescence imaging.

### Auxin degron assay

Cells were grown for 5 h on YM2 minimal medium containing glucose and then inoculated to OD_600_ = 0.05 overnight in the same fresh medium. The next morning cultures were treated with (+) or without (−) indole-3-acetic acid (auxin, Sigma-Aldrich, I3750) for up to 5 h. Auxin was added to a final concentration of 500 μM.

### Image acquisition

Live cells were analyzed with an Axiovert 200M microscope (Carl Zeiss, Inc.) equipped with Exfo X-cite 120 excitation light source, band-pass filters (Carl Zeiss, Inc. and Chroma), and a Plan-Fluar 100×/1.45 NA or A-Plan 40×/0.65 NA Ph2 objective lens (Carl Zeiss, Inc.) and a digital camera (Orca ER; Hamamatsu). Image acquisition was performed using Volocity software (Perkin-Elmer). Fluorescence images were routinely collected as 0.5-mm Z-Stacks and merged into 1 plane after contrast enhancing in Volocity and processed further in Photoshop (Adobe) where only the level adjustment was used. On occasion (as indicated in the text) images were collected as single-plane images. Bright-field images were collected in 1 plane. Auxin strains that were adenine deficient were mated with BY4741 or BY4742 derivatives and diploids selected to circumvent auto-fluorescence caused by the lack of adenine.

## Supplementary Material

Additional material

## Abbreviations:

Aid: auxin-inducible degron
Atg: autophagy-related
HA: hemagglutinin
PA: protein A
PAS: phagophore assembly site
PTS1: peroxisomal targeting signal type 1
