# Supplementary material for: Deficiency of the exportomer components Pex1, Pex6, and Pex15 causes enhanced pexophagy in Saccharomyces cerevisiae
Source: Autophagy. 2014 Mar 18;10(5):835–45. doi: 10.4161/auto.28259 (PMC5119063; doi:10.4161/auto.28259)
Supplement: Additional material [file kaup-10-05-10928259-s001.zip › 2013AUTO0610R1-Sup.pdf]

Supplemental Material to:

James M Nuttall, Alison M Motley, and Ewald H Hettema

Deficiency of the exportomer components Pex1, Pex6, and Pex15 causes enhanced pexophagy in *Saccharomyces cerevisiae*

Autophagy 2014; 10(5)
http://dx.doi.org/10.4161/auto.28259

www.landesbioscience.com/journals/autophagy/article/28259

Figure S1

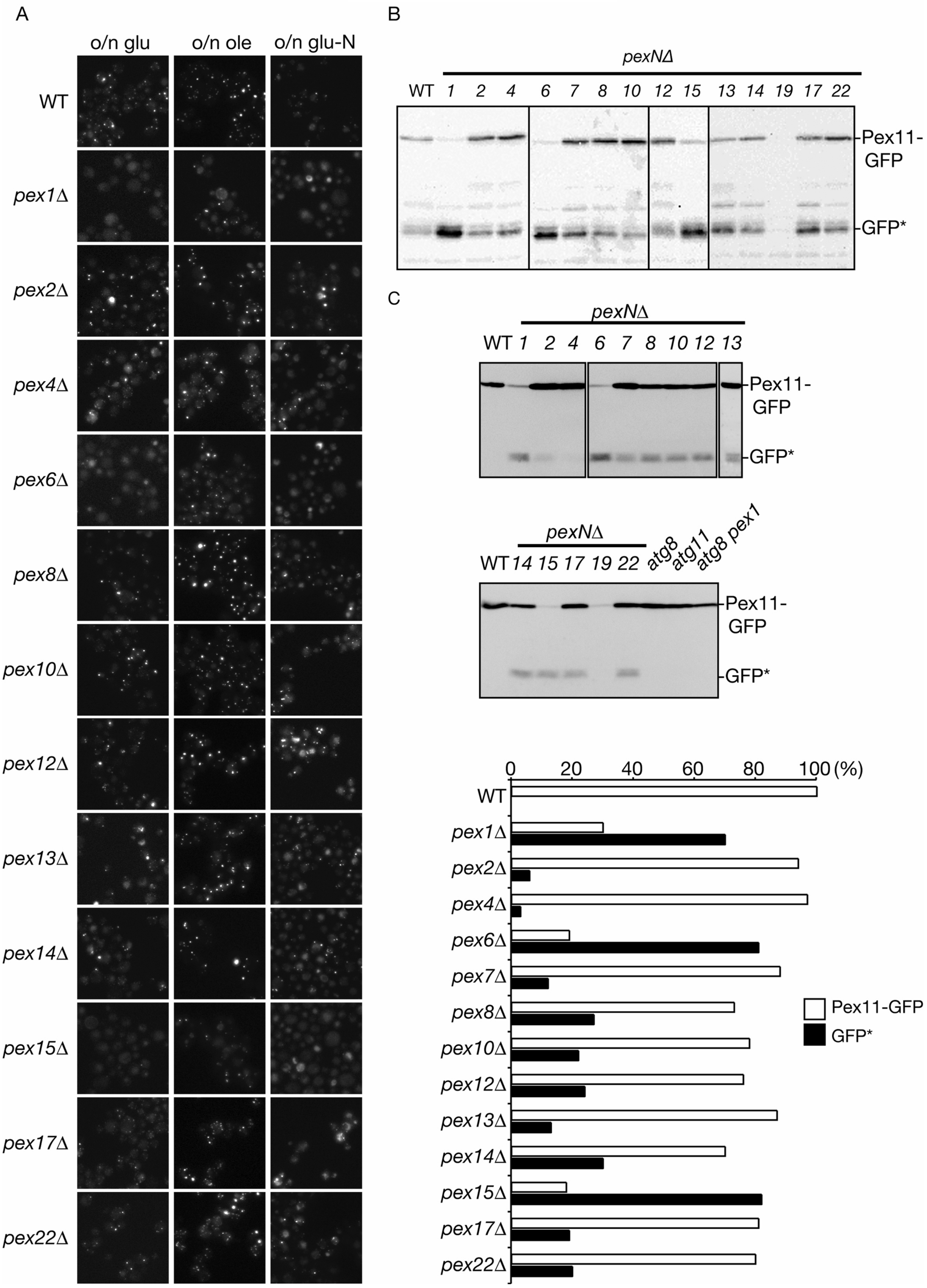

A
o/n glu
o/n ole
o/n glu-N
WT
pex1Δ
pex2Δ
pex4Δ
pex6Δ
pex8Δ
pex10Δ
pex12Δ
pex13Δ
pex14Δ
pex15Δ
pex17Δ
pex22Δ
B
pexNΔ
WT 1 2 4 6 7 8 10 12 15 13 14 19 17 22
Pex11-GFP
GFP*
C
pexNΔ
WT 1 2 4 6 7 8 10 12 13
Pex11-GFP
GFP*
pexNΔ
WT 14 15 17 19 22 atg8 atg11 atg8 pex1
Pex11-GFP
GFP*
0 20 40 60 80 100 (%)
WT
pex1Δ
pex2Δ
pex4Δ
pex6Δ
pex7Δ
pex8Δ
pex10Δ
pex12Δ
pex13Δ
pex14Δ
pex15Δ
pex17Δ
pex22Δ
Pex11-GFP
GFP*

Figure S2

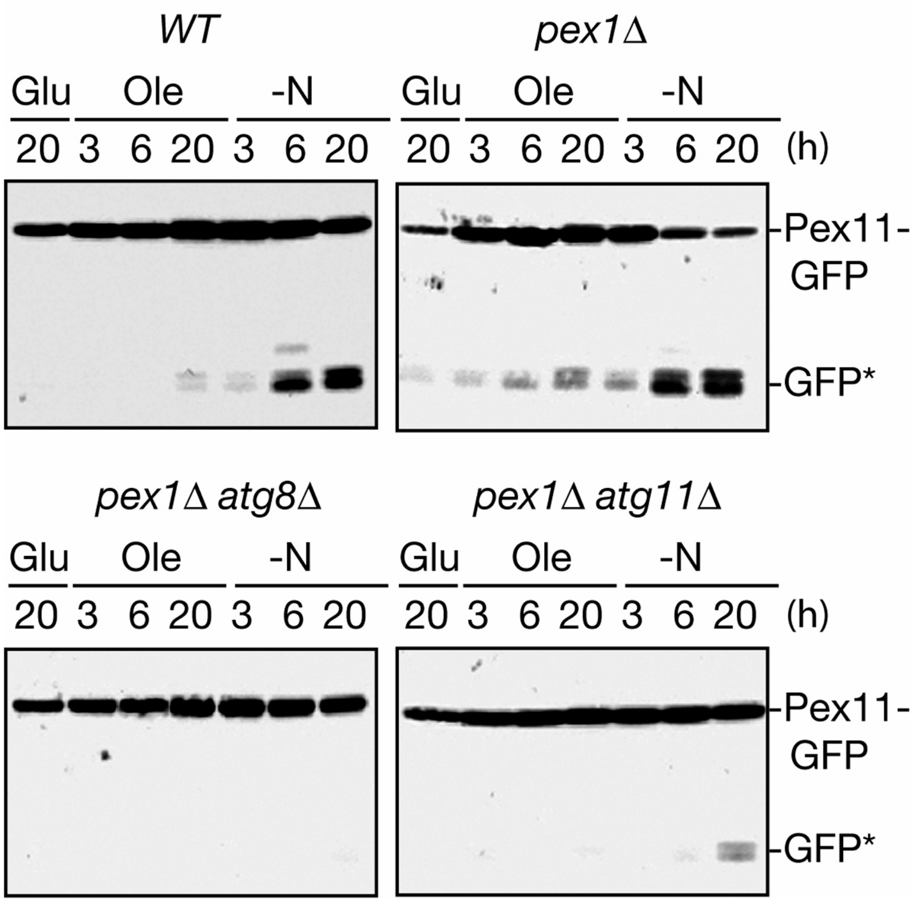

WT
pex1Δ
Glu
Ole
-N
20 3 6 20 3 6 20 (h)
-Pex11-GFP
-GFP*
pex1Δ atg8Δ
pex1Δ atg11Δ

Figure S1. *pex1Δ*, *pex6Δ,* and *pex15Δ* cells show increased breakdown of peroxisomal membranes. (**A**) Twelve mutants in peroxisomal matrix protein import were examined for pexophagy under the indicated growth conditions using Pex11-GFP. Immunoblot analysis with monoclonal anti-GFP antibody shows *pex1Δ*, *pex6Δ,* and *pex15Δ* have increased pexophagy after nitrogen starvation (**B**) and post-logarithmic growth on glucose (**C**). For nitrogen starvation, cells were grown on oleate medium and shifted to SD-N medium. Samples were harvested after 20 h (**B**). The full-length and breakdown product signals in post-logarithmic cultures (**C**) were quantified in ImageJ and expressed as a percentage of total signal.

Figure S2. Peroxisomal membrane breakdown in *pex1Δ* cells is enhanced under various growth conditions and is dependent on Atg8 and Atg11. Immunoblot analysis of Pex11-GFP-expressing cells that were grown on glucose-containing medium for 20 h, transferred to oleate medium (for 3, 6, 20 h), then shifted to SD-N medium (-N) (for 3, 6, 20 h). Immunoblotting was done with monoclonal anti-GFP antibody.
